# Quantitative Assessment of Hydrogel Printability in Extrusion Bioprinting

**DOI:** 10.3390/gels12030189

**Published:** 2026-02-24

**Authors:** Shengkai Yu, Yang Luo, Shang Chen, Jiashuo Fan, Hua Zhang

**Affiliations:** Research Institute of Smart Medicine and Biological Engineering, Health Science Center, Ningbo University, Ningbo 315211, China

**Keywords:** 3D bioprinting, hydrogel, printability, quantitative assessment

## Abstract

Extrusion-based 3D bioprinting enables the fabrication of complex tissue structures, yet achieving both high structural fidelity and cell viability remains challenging due to complex bioink rheology and parameter interplay. This review presents a quantitative framework linking hydrogel properties to printing outcomes. Key rheological features—shear-thinning, yield stress, reversible gel-sol transition, self-healing, and creep resistance—are examined for their roles in extrusion and shape retention. We also evaluate printing accuracy using metrics such as filament uniformity and multilayer stability. Advanced models, including Herschel-Bulkley and extrusion pressure models, correlate material parameters with flow dynamics and predict critical factors like wall shear stress. Finally, we propose an integrated assessment system combining material properties, process parameters, and structural fidelity to guide bioink design and printing optimization, advancing the field of hydrogel bioprinting.

## 1. Introduction

Three-dimensional bioprinting has emerged as a transformative biofabrication technology that enables the precise construction of complex volumetric tissue architectures. By leveraging computer-aided design and manufacturing principles, this layer-by-layer additive process facilitates the spatially controlled deposition of cell-laden bioinks to create tissue constructs with well-defined geometries and biological functionalities. The principal advantage of 3D bioprinting lies in its unique capacity to replicate the compositional complexity and spatial organization of native extracellular matrices, thereby creating unprecedented opportunities for developing physiologically relevant in vitro models, implantable tissue equivalents, and advanced drug screening platforms [1,2]. These capabilities hold considerable promise across a broad spectrum of biomedical applications, including regenerative medicine, disease modeling, pharmaceutical development, and soft robotics [3,4].

Among available bioprinting modalities, extrusion-based bioprinting has attracted significant interest owing to its distinct advantages. This approach demonstrates broad compatibility with diverse biomaterials spanning natural and synthetic polymers. Such versatility enables the formulation of bioinks capable of in situ solidification through physical or chemical crosslinking mechanisms, thereby expanding the potential for fabricating functional tissue constructs [5]. Furthermore, through precise modulation of process parameters such as extrusion pressure, printing path, and temperature, this technique can be adapted to engineer tissues across a spectrum of scales and mechanical properties, ranging from capillary networks to cartilaginous and osseous structures. Importantly, in contrast to light-based bioprinting techniques that may introduce phototoxicity risks, the comparatively gentle extrusion process better preserves cell viability and functionality, which constitutes an essential prerequisite for generating metabolically active, long-lasting tissue constructs [6,7].

However, the simultaneous realization of high structural fidelity and excellent cell viability remains a fundamental challenge within the field. This challenge originates from a complex interplay of multiple constraints. Bioinks must exhibit carefully tailored rheological properties to successfully navigate the printing process. During extrusion, they must demonstrate pronounced shear-thinning behavior to enable smooth flow through microscale nozzles while minimizing shear-induced cell damage [8,9,10]. Upon deposition, they must rapidly either recover their mechanical modulus through self-healing mechanisms or undergo rapid gelation to maintain the as-deposited filament morphology and support the weight of subsequent layers, thereby necessitating adequate yield stress and creep resistance. Concurrently, printing parameters encompassing nozzle diameter, extrusion rate, printing speed, and crosslinking strategy require meticulous optimization to align with the rheological characteristics of the bioink. Any discrepancy may result in printing failures such as nozzle obstruction, filament discontinuity, pore coalescence, or structural collapse [11,12].

The multifaceted nature of these challenges highlights the limitations of conventional qualitative assessment methods, which have proven insufficient for the rational design of high-performance bioinks and the optimization of printing processes [13,14]. The establishment of a systematic and quantitative framework for evaluating bioink printability has therefore become indispensable for bridging the gap between material development and successful printing outcomes. Such a framework should integrate multiple assessment dimensions: first, comprehensive rheological characterization to quantify how critical parameters including shear-thinning behavior, yield stress, modulus recovery kinetics, and gelation dynamics govern ink extrusion behavior and structural stability [15]; second, quantitative microstructural analysis to evaluate morphological metrics such as filament uniformity, printed grid pore regularity, and dimensional fidelity of multilayered structures; and third, theoretical modeling to establish predictive relationships between material properties, process parameters, and printing outcomes [16,17].

This review aims to provide a comprehensive examination of quantitative assessment methodologies for micro-extrusion hydrogel bioink printability, with particular emphasis on establishing quantitative structure-property relationships between rheological parameters and printing morphology. It will address recent advances in implementing mathematical models such as the Herschel-Bulkley model to predict bioink flow behavior during printing, and discuss emerging strategies for integrating experimental characterization with computational approaches [18]. Investigating the influence of printing parameters is essential for understanding the overall impact on extrusion-based bioprinting and predicting optimal parameters. The successful implementation of extrusion-based bioprinting relies on the synergistic optimization of multiple parameters, necessitating the establishment of a comprehensive evaluation framework that integrates rheological properties, structural fidelity, and biological performance. Recognizing the current paucity of quantitative investigations into hydrogel-based bioinks, we propose a systematic strategy to quantitatively evaluate the influence of key printing parameters on extrusion-based hydrogel printing, thereby establishing a rational framework for the optimization of 3D printing parameters. The ultimate objective is to provide a robust theoretical foundation and methodological guidance for the rational design of bioinks, quantitative optimization of printing processes, and reliable fabrication of complex, highly viable tissue structures, thereby accelerating the translation of bioprinting technologies toward clinical applications.

## 2. Quantitative Evaluation of Rheological Properties of Hydrogels

**Figure 1:**
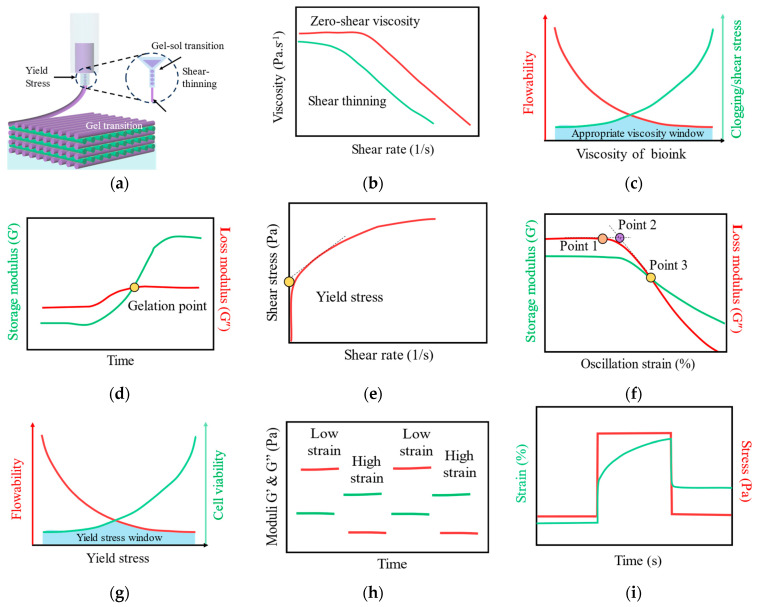
Quantitative evaluation of rheological properties for extrusion-based printing hydrogels. (**a**) Illustration of the micro-extrusion bioprinting process of hydrogel bioinks and its correlation with rheological properties. (**b**) Shear-rate sweep curves quantifying the viscosity and shear-thinning behavior of the bioink. (**c**) Schematic highlighting the critical balance between preserving structural fidelity and avoiding printhead clogging or excessive shear stress. (**d**) Relationship between yield stress and both flowability and cell viability. (**e**,**f**) Determination of yield shear stress through shear sweeps (**e**) and strain sweeps (**f**) for hydrogel precursor solutions and dynamically cross-linked gels, respectively. (**g**) Schematic highlighting the relationship between yield stress, flowability, and cell viability. (**h**) Self-healing assessment of bioinks using alternating strain tests. (**i**) Representative creep and recovery curves of the bioinks.

The successful implementation of micro-extrusion bioprinting critically depends on precise control over the viscoelastic properties of hydrogel bioinks [19,20]. Unlike simple Newtonian fluids, hydrogels exhibit a complex interplay between viscous (liquid-like) and elastic (solid-like) behaviors, which governs their performance throughout the printing process. Table 1 summarized the key rheological properties of several representative printable hydrogel bioinks. These properties dictate essential aspects ranging from extrusion behavior through fine nozzles and shape retention upon deposition to structural stability during multilayer stacking (Figure 1a). A fundamental understanding of core viscoelastic parameters is therefore indispensable for achieving high printing fidelity. This section systematically examines five key rheological characteristics that define hydrogel behavior during bioprinting, establishing a comprehensive framework for rational bioink design and process optimization [21].

### 2.1. Viscosity and Shear-Thinning Behavior

Viscosity, defining a fluid’s internal resistance to flow, is a pivotal rheological parameter in bioprinting that directly governs bioink extrusion dynamics [41]. It critically influences not only the material’s flow behavior but also determines the morphological precision and structural integrity of printed constructs. Excessively high viscosity necessitates greater extrusion pressure, increasing the risks of nozzle clogging and detrimental shear stress on encapsulated cells. Conversely, insufficient viscosity leads to poor shape retention, causing filament breakup or collapse, thereby compromising the intended 3D architecture [42,43]. Consequently, comprehensive evaluation and then precise optimization of a hydrogel bioink’s viscosity are essential by using a shear-rate sweep (Figure 1b). A critical comparison of different formulation strategies reveals that achieving optimal viscosity is not a matter of simply increasing polymer concentration. For instance, in a gelatin/alginate semi-crosslinked system, printability is critically dependent on gelatin concentration: formulations with 15% gelatin demonstrate optimal shear-thinning behavior, a spreading ratio closest to the ideal value of 1, and superior shape fidelity [44]. In stark contrast, formulations with higher gelatin concentrations exhibited significant inhomogeneity, leading to a high standard deviation in extrusion force and discontinuous printing, thereby compromising printing accuracy. This comparison underscores that while increasing concentration is a common strategy to enhance viscosity, it may result in diminishing returns or even failure if it leads to material inhomogenization. In contrast, a formulation containing 20% gelatin exhibits significant inhomogeneity, resulting in high standard deviations in extrusion force and discontinuous extrudability, which compromises printing precision. This underscores the crucial importance of systematically balancing ink composition to achieve smooth flow and high structural fidelity (Figure 1c).

Hydrogel precursors frequently exhibit pronounced non-Newtonian characteristics, with shear-thinning behavior—where apparent viscosity decreases under increasing shear rate—being particularly advantageous [45]. This property enables bioinks to flow readily under high-shear conditions within the nozzle, yet rapidly recover viscosity upon deposition to maintain filament shape. The physical mechanism stems from shear-induced reorganization of polymer networks: shear forces cause chain disentanglement and alignment, reducing intermolecular friction and thus lowering viscosity [46,47]. While effective for basic extrusion, this purely physical mechanism offers limited control over recovery kinetics and long-term stability, representing a significant constraint compared to more sophisticated chemical approaches. The employment of dynamic cross-linking emerges as an alternative strategy to circumvent this limitation, in stark contrast to traditional physical entanglement. For example, a hyaluronic acid-based hydrogel incorporating dual dynamic cross-linking—through rapid thiazolidine formation and slow disulfide exchange—utilizes differential reaction kinetics as a built-in rheological regulator, where fast thiazolidine bonds provide immediate post-extrusion stability while reversible disulfide bonds enable gradual network strengthening and autonomous self-healing. Upon cessation of shear, disulfide exchange recommences, restoring structural integrity and mechanical properties such as the storage modulus. Moreover, cell-secreted glutathione can further modulate the disulfide network, offering a dynamically responsive and cell-remodelable platform for designing bioinks that balance printability, stability, and biological interactivity [48].

### 2.2. Liquid-Solid Transition and Gelation Kinetics

The sol-gel transition, fundamentally governed by gelation mechanism and kinetics, is critical for prompt shape retention upon extrusion. This process is characterized by dynamic changes in viscoelastic moduli, commonly monitored via in situ rheology. Initially, the storage modulus (G′) remains lower than the loss modulus (G″), reflecting liquid-like behavior. As crosslinking progresses, G′ gradually increases and eventually surpasses G″, with their intersection defined as the gel point, marking the formation of a solid-like network (Figure 1d).

Multiple factors influence the sol-gel transition rate, including the conditions required to initiate crosslinking and the reaction kinetics of the functional groups. For example, Mooney et al. [49] developed develop a new class of injectable tissue adhesives by leveraging the dynamic crosslinking chemistry of Schiff base reactions, which exhibited a gelation time of 60 min and a polymer solid content of up to 10 wt%. However, for applications such as 3D bioprinting, hydrogel precursors must undergo rapid gelation to enable prompt shape retention upon extrusion.

To enhance the sol-gel transition, several strategies have been explored, including the introduction of catalysts, structural modification of crosslinked Herschel-Bulkleys, and the construction of dual or multiple crosslinking networks [50,51]. A critical comparison of these strategies reveals the inherent trade-offs between their efficiency, safety, and complexity. For instance, Lou et al. [52] tackled the issue of excessive cellular shear damage resulting from slow gelation in dynamic hydrazone-crosslinked hyaluronic acid by designing two water-soluble organic catalysts that modulate hydrazone bond formation kinetics. Their findings revealed that although the equilibrium modulus was largely preserved, the gelation time was markedly reduced from over 1000 s to 180 s [53,54]. Moreover, the hydrogels incorporating catalysts demonstrated improved stress relaxation properties [55,56]. It should be noted, however, that the potential leaching of water-soluble catalysts from the hydrogel network may raise concerns regarding cytotoxicity and immunogenicity, thereby limiting their utility in tissue repair applications.

To circumvent the need for catalysts and simplify the synthetic process, Chen et al. [57] developed a catalyst-free, rapid, and biocompatible hydrogel crosslinking strategy based on the condensation reaction between o-phthalaldehyde (OPA) and N-nucleophiles (amine, hydrazide, and aminooxy groups). During cross-linking, OPA reacts with amines (or hydrazides) spontaneously and chemoselectively under physiological conditions, forming stable phthalimidine (or reversible hydrazone) linkages. Moreover, strong adhesion of the hydrogel to tissues can be achieved via the spontaneous coupling of OPA with amine groups present in tissues (typically within extracellular matrix proteins), resulting in stable phthalimidine bonds [58]. This design not only addresses the gelation rate issue but also additionally confers tissue adhesiveness, embodying a synergistic advantage in chemical design.

In addition to covalent catalysis or ionic complexation, physical interactions offer an alternative pathway to facilitate gelation kinetics. For instance, Han et al. [59] introduced a catalyst-free strategy to accelerate the sol-gel transition by leveraging electrostatic interactions [60]. They synthesized water-soluble methacryloyl chitosan and aldehyde-modified hyaluronic acid from cationic chitosan and anionic hyaluronic acid. By synergistically combining dynamic Schiff base bonding between amino and aldehyde groups with electrostatic interactions between amino and carboxyl groups, they achieved rapid hydrogel formation within just 5 s. To address the challenge of balancing rapid gelation with long-term stability in bioprinting, our team developed a micelle-mediated dynamic hyaluronic acid hydrogel [21]. By incorporating self-assembled F127DA nanomicelles into a dynamic network formed from hydrazide and aldehyde-functionalized hyaluronic acid, we utilized hydrogen bonding and hydrophobic interactions to drastically shorten the gelation time from 208 s to 3 s, while simultaneously enhancing network stability through macromolecular crosslinking. This approach enabled low-shear mixing-injection bioprinting with high cell viability and supported the fabrication of a biomimetic skin construct with well-defined epidermal and dermal layers. This finding provides compelling evidence that the rational integration of multiple crosslinking mechanisms—namely dynamic covalent bonds, hydrogen bonding, and hydrophobic interactions—offers a synergistic solution to the challenges of gelation kinetics, biocompatibility, and long-term stability, which remain unattainable through any single strategy alone.

### 2.3. Yield Stress

**Figure 2:**
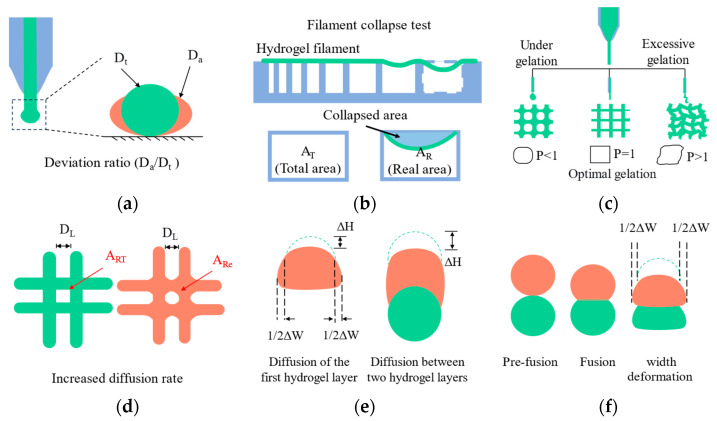
Structural analysis of hydrogel 3D printability. (**a**) Deviation ratio of hydrogel deformation during printing [72]. (**b**) Filament collapse angle and spreading factor illustrating the balance between viscosity and surface tension during initial deposition [73]. (**c**) Printability (*P*) evaluated under three typical gelation statuses, namely under-, proper- and over-gelation. (**d**) Schematic of width deformation (Δ*W*) in the first hydrogel layer and deformation of the second layer, including its fusion with the first layer [74]. (**e**) Horizontal diffusion of the first hydrogel layer and two hydrogel layers, along with vertical fusion progression between two layers [74]. (**f**) Comparative analysis of theoretical and actual printed grid structures [74]. (**a**) reprinted from [72], under the CC BY 4.0 license. (**b**) reprinted from [73], under the CC BY 4.0 license. (**d**–**f**) reprinted from [74], under the CC BY 4.0 license.

The yield stress is defined as the critical stress value required to transition a fluid from a static state to a flowing state. In 3D bioprinting, this parameter plays a crucial role in maintaining the structural integrity of hydrogel bioinks while effectively protecting encapsulated cells from shear-induced damage during the extrusion process [61]. For hydrogel precursor solutions, a continuously increasing shear rate is applied from low to high values, and the corresponding shear stress is measured. Fluids with a yield stress must overcome a minimum stress threshold to initiate flow. On the stress-rate curve, this manifests as a distinct plateau or inflection point (Figure 1e). For gels, their yield stresses are commonly characterized using three principal rheological approaches: (1) defining the yield point as the critical stress marking the end of the linear viscoelastic region (Point 1) (Figure 1f); (2) identifying the yield stress as the shear stress at which the storage modulus (G′) and loss modulus (G″) intersect in the nonlinear regime (Point 3); and (3) for hydrogels that display stress overshoot behavior, taking the peak value of G′ or G″ observed during a stress amplitude sweep as the critical yield point (Point 2). The yield stresses determined by these methods generally differ in magnitude: the G′ - G″ crossover point typically gives the highest value, followed by the stress overshoot peak, while the end point of the linear viscoelastic region yields the lowest value. Moreover, yield stress demonstrates pronounced frequency dependence. With increasing test frequency, the linear viscoelastic region becomes narrower, and the measured yield stress correspondingly decreases.

To regulate the yield stress of hydrogel systems during printing, functional additives are commonly incorporated into the formulation for modulation, such as Pluronic F127 (Sigma-Aldrich, Milan, Italy), gellan gum (Sigma-Aldrich, Italy), hyaluronic acid (Tsinghua University, Beijing, China), carrageenan (Sigma-Aldrich, Italy), and various inorganic nanofillers [62,63]. For example, Deo et al. [36] designed a biphasic granular colloidal bioink to optimize both cell viability and printing fidelity. This composite ink features cell-laden polyethylene glycol (PEG) hydrogel microparticles uniformly dispersed within a continuous gelatin methacryloyl (GelMA)-nanosilicate colloidal matrix. The introduction of nanosilicates and PEG microgels yielded a granular colloidal hydrogel exhibiting pronounced shear-thinning behavior. Compared to its colloidal hydrogel counterpart, this granular system demonstrates a reduced yield stress, which promotes smoother extrusion and enhances post-printing cell survival. Concurrently, the yield stress remains substantially higher than that of pure GelMA, thereby ensuring superior printing resolution and structural stability Figure 1g) [64,65,66]. This strategy of achieving decoupled regulation of yield stress—namely, simultaneously attaining low extrusion resistance and high shape fidelity—through the construction of multiphase systems remains unattainable in single-component systems and represents a significant direction for advancement in bioink design.

### 2.4. Reversible Gel-Sol Transition and Self-Healing Properties

The reversible gel-sol transition of hydrogels represents another critical consideration in the design of high-performance bioinks. Mediated by non-covalent interactions or dynamic covalent bonds, this phase-change behavior enables controlled switching between gel and sol states under external stimuli such as shear stress, temperature, or light, thereby effectively reconciling the intrinsic trade-off between “flowability and formability” in bioprinting. Accurate characterization of the gel-sol transition behavior relies on systematic rheological evaluation methods. Among these, the three-interval thixotropy test most closely mimics the actual printing process (Figure 1h): the first interval measures the initial modulus within the linear viscoelastic region to assess the static structural strength of the ink; the second interval applies high strain to simulate the extrusion process and monitors the gel-sol transition and shear-thinning extent; the third interval restores low-strain conditions to track the temporal evolution of the modulus, quantifying the structural recovery kinetics of the ink—a key indicator for evaluating its self-healing capability and structural fidelity [67]. A critical comparison of various rheological characterization methods reveals that the three-interval thixotropy test is regarded as the “gold standard” because it captures both the sequential processes of shear-induced liquefaction and subsequent recovery of solid-like properties. In contrast, conventional steady-state shear or dynamic oscillatory tests can only assess behavior under individual states separately, failing to reflect the dynamic evolution of ink states during the printing process.

The value of the reversible gel-sol transition in bioprinting is manifested in three key aspects. First, this property confers excellent extrusion performance to bioinks. When subjected to high shear forces within the printing nozzle, the ink undergoes a transition from a gel to a sol state, exhibiting marked shear-thinning behavior that drastically reduces viscosity [68]. This allows smooth extrusion under low pressure, effectively minimizing mechanical damage to encapsulated cells. Upon exiting the nozzle and removal of shear, the ink rapidly reverts to the gel state, achieving instantaneous self-healing [35]. This rapid recovery is essential for maintaining the shape fidelity of extruded filaments and preventing structural collapse, thereby ensuring accurate fabrication of complex 3D architectures. Second, this reversible transition provides an ideal carrier environment for cells. In the sol state, cells reside in a low-viscosity, low-shear stress milieu, whereas upon re-gelation, cells are immediately provided with a stable 3D support that mimics the mechanical and biological functions of the native extracellular matrix. Furthermore, this functionality enables advanced printing strategies, such as the “penetration–self-healing” mode in suspension bioprinting, or the precise construction of multi-material and chemical gradients via localized stimulation, opening new avenues for engineering complex tissue constructs with spatial heterogeneity [5,69]. From the perspective of technological advancement, this printing strategy based on reversible phase transitions transcends the limitations of conventional layer-by-layer deposition, elevating bioprinting from mere shape replication to a new stage of functional construction capable of simulating the dynamic microenvironment of native tissues.

### 2.5. Creep Behavior

The creep behavior of hydrogels represents a critical determinant for the successful fabrication of stable three-dimensional structures. Creep refers to the time-dependent increase in strain under constant stress. This phenomenon carries substantial practical implications for 3D bioprinting: following deposition, each layer of hydrogel must sustain not only its own weight but also the cumulative load of subsequently printed layers. Insufficient resistance to creep deformation can lead to gradual, irreversible structural changes during or after the printing process, potentially resulting in collapse, pore closure, or overall dimensional shrinkage—ultimately compromising structural fidelity and geometric accuracy. Therefore, systematic evaluation of creep and creep recovery behavior is essential for predicting and ensuring the structural stability of hydrogels as bioinks in the post-printing phase [70].

Accurate characterization of creep behavior relies on standardized rheological creep testing. This testing is typically conducted in transient mode: a small, constant stress—maintained within the linear viscoelastic region to prevent microstructural damage—is applied to the hydrogel sample. The resulting strain evolution over time is monitored to generate a compliance-time curve. Upon stress removal, the subsequent strain recovery is tracked to assess the material’s recovery capability. Compliance, defined as the reciprocal of modulus (strain/stress), serves as a direct indicator of the material’s deformability. Lower compliance values signify greater resistance to deformation under applied stress, typically corresponding to superior anti-creep performance (Figure 1i). The classic four-element Burgers model is widely adopted because it deconstructs complex creep behavior into three components with distinct physical meanings. In contrast, simpler models such as Maxwell or Kelvin–Voigt can only describe singular processes, thereby failing to capture the comprehensive mechanical response of hydrogels under sustained loading.

To gain deeper insight into the physical mechanisms underlying creep behavior, experimental data are commonly fitted using the classical four-element Burgers viscoelastic model. The model is expressed as *J_e_(t) = J*_0_ *+ J*_1_ [1 *− exp*(*−t/τ*)] *+ t/η*, which comprehensively describes three fundamental material responses: *J*_0_ represents the instantaneous compliance contributed by immediate elastic deformation (Maxwell spring), corresponding to the initial elastic response of a printed layer under sudden loading; the term *J*_1_[1 − *exp*(*-t/τ*)] describes the delayed compliance arising from viscoelastic relaxation (Kelvin–Voigt unit), where *τ* denotes the retardation time, reflecting the rearrangement and relaxation kinetics of hydrogel polymer chains under stress; finally, the *t/η* term accounts for viscous flow, with *η* representing viscosity, which reveals irreversible plastic deformation and serves as the primary contributor to long-term structural instability. This modeling approach enables quantitative analysis of the respective contributions of instantaneous elasticity, delayed elasticity, and viscous flow, thereby providing crucial theoretical guidance for optimizing bioink formulations to suppress undesirable viscous flow.

For example, Zhao et al. [71] demonstrated that untreated polyvinyl alcohol (PVA) (Sinopharm Chemical Reagent Co., Ltd., Shanghai, China)/chitosan (CS) (Jinhu Company, Shanghai, China) hydrogels exhibit high compliance and irreversible deformation in creep tests, indicating insufficient long-term stability under sustained loading when used as coating materials. In contrast, the HPVA/CS-PSPMA (SPMA were purchased from TCI Co., Ltd., Shanghai, China) hydrogel reinforced through a multi-level crystallization strategy demonstrates higher mechanical strength, good anti-swelling behavior in physiological media, and typical creep-recovery properties. Under high contact pressure (2.0–3.0 MPa) in PBS, HPVA/CS-PSPMA shows favorable lubrication performance (coefficient of friction, COF: 0.02–0.03). Remarkably, under severe friction test conditions (100 k cycles, P ≈ 2.5 MPa), HPVA/CS-PSPMA maintains a lower and more stable COF (≈0.027) compared to natural bovine cartilage, with negligible surface wear. More importantly, after friction testing, the mechanical deformation in the sliding contact area of the HPVA/CS-PSPMA composite recovers to a large extent.

The HPVA/CS-PSPMA material thus exhibits excellent lubricity, robustness, wear resistance, and mechanical recovery properties. This multi-level reinforcement strategy enables a performance transition from “high creep, low recovery” to “low creep, high recovery,” providing a critical theoretical foundation and design paradigm for engineering high-performance bioinks capable of withstanding transient loads during printing while maintaining structural stability over long-term service.

## 3. Structural Analysis of Hydrogel 3D Printability

Rheological characterization predicts hydrogel printability, yet a persistent challenge lies in the precise translation of these properties into consistently high-fidelity printed structures. To address this gap, structural analysis of hydrogel 3D printability is essential. This section moves beyond ink-centric measurements to conduct a systematic, quantitative evaluation of the printed constructs themselves. By examining metrics such as filament uniformity, pore geometry, layer adhesion, and overall dimensional accuracy, we establish objective criteria to compare bioinks and optimize printing parameters. This analysis provides the critical feedback loop needed to close the gap between predicted printability and actual printing outcome.

### 3.1. Filament Morphological Uniformity and Dimensional Fidelity

The fundamental building block of bioprinted structures is the extruded filament, whose morphological uniformity is critical for determining printing resolution and the structural integrity of the final scaffold. Ideal filaments exhibit circular cross-sections with consistent diameter and smooth surfaces, while practical printing often produces variations due to material properties and process parameters. To objectively assess these morphological features, key quantitative metrics are employed, including filament diameter consistency, surface roughness, and cross-sectional geometry. A primary indicator of extrusion stability is the diameter deviation ratio (D_a_/D_t_), where D_a_ is the measured filament diameter and D_t_ is the theoretical diameter calculated from the nozzle size and flow rate (Figure 2a) [72]. A ratio approaching 1.0 reflects optimal material deposition with minimal die-swelling effects. While a filament diameter deviation ratio approaching 1.0 is generally regarded as ideal, an exclusive focus on this value may obscure more complex material behaviors. For instance, inks with pronounced shear-thinning properties may achieve a near-1.0 ratio even under low extrusion pressure, yet their resistance to collapse across spanning regions often falls substantially below that of systems which exhibit slight swelling but possess a denser network architecture. Therefore, this static metric must be integrated with dynamic mechanical assessments to enable a comprehensive evaluation of printing fidelity.

Beyond static dimensional analysis, the filament collapse test evaluates a hydrogel’s ability to maintain structural shape under spanning conditions. In this assay, filaments are deposited over a series of pillars with progressively increasing inter-pillar spacing (Figure 2b) [73]. Greater spans typically lead to increased deflection or collapse. To quantify this behavior, the hydrogel is extruded across the pillar array, and the deposited structure is imaged. The theoretical area (*A_t_*) enclosed by adjacent pillars is compared with the actual deposited area (*A_r_*) using image analysis software (ImageJ 1.47). The collapse rate is then calculated as:

(1) $$Collapse\;Rate=\frac{A_{t}\;-\;A_{r}}{A_{t}}\times 100\%,$$

A collapse rate of 0% indicates perfect shape retention with no sagging, whereas higher values reflect increasing structural instability. This test provides a quantitative, comparable measure of a hydrogel’s mechanical robustness during the critical initial deposition phase, enabling systematic evaluation and optimization of both bioink formulations and printing parameters [72,75].

### 3.2. Grid Structure Regularity and Printing Accuracy Assessment

Following the assessment of filament uniformity and collapse stability, the evaluation of printability further extends to the regularity and geometric accuracy of the assembled lattice or grid structures. The morphological variations of extruded filaments and their stacked architectures at both micro- and macroscopic scales form the quantitative basis for evaluating the quality of printed structures. An optimally cross-linked hydrogel should maintain well-defined filament morphology during printing and form a regular three-dimensional grid that closely matches the intended design, such as square or rectangular pores (Figure 2c) [76]. When gelation is insufficient, the extruded ink behaves in a liquid-like manner, often resulting in layer fusion and the formation of approximately circular or oval pores [77,78]. Conversely, excessive gelation can cause filament distortion under extrusion stress, leading to irregular pore sizes within the grid. To semi-quantitatively assess the influence of gelation characteristics on printing performance, the roundness (*C*) of grid pores can be measured and calculated as follows:

(2) $$C=\frac{4\pi A}{L^{2}}$$

where *L* is the perimeter of the closed pore (μm) and *A* is its area (μm^2^). Geometrically, a perfect circle has a roundness of 1, while a perfect square has a roundness of π/4. Thus, a *C* value closer to 1 indicates a more circular pore shape, whereas a value closer to π/4 suggests a pore structure approximating a square. Based on this metric, the printability index (*P*) of a bioink can be defined as:

(3) $$P=\frac{\pi }{4}\cdot \frac{1}{C}=\frac{L^{2}}{16A}$$

The *P* value reflects the gelation state of the hydrogel ink during printing: *P* < 1 indicates under-gelation, *P* > 1 indicates over-gelation, and *P* = 1 corresponds to the optimal gelation state for printing (Figure 2c).

Using this analytical framework, Rency Geevarghese et al. [28] systematically evaluated the printability of alginate/carboxymethyl cellulose/gelatin methacryloyl (Alg/CMC/GelMA) composite hydrogels. When printed at 37 °C, these hydrogels exhibited distinct filament morphology and uniform square-shaped grid structures. Through systematic optimization of component concentrations, it was found that hydrogels with optimal printability exhibited *P* index values ranging from 0.9 to 1.1, while the fiber diameter closely matched the nozzle size (200 µm). Specifically, with fixed concentrations of Alg and CMC (4% Alg–10% CMC), increasing the GelMA content from 8% to 12% and 16% resulted in a gradual decrease in extrusion pressure (from 185 kPa to 165 kPa), while *P* values remained stable between 0.93 and 0.95. By carefully balancing composition, temperature, and crosslinking strategy, the Alg/CMC/GelMA hydrogel system achieves excellent printability, mechanical stability, and biocompatibility, making it a promising candidate for gradient tissue engineering applications.

### 3.3. Stability Analysis of Multi-Layer Stacked Structures

In layer-by-layer additive manufacturing, the quality of 3D hydrogel structures depends not only on the morphological fidelity of individual filaments but also on the integrated structural evolution during multi-layer stacking. Interlayer fusion behavior, creep-induced collapse under gravity, and the cumulative dimensional deviations across layers collectively determine the final geometric accuracy and long-term stability of the fabricated constructs.

Printed hydrogel structures are susceptible to fusion, spreading, and collapse under gravitational forces, where the layer height is directly influenced by filament height deformation Δ*H* (Figure 2d) [74]. Vertical merging between adjacent hydrogel layers further compromises the structural resolution, with the extent of fusion governed by the interlayer height difference Δ*h* (Figure 2e) [74]. During collapse and merging, the width difference ΔW in the horizontal direction tends to increase. Single-layer filaments undergo gravitational creep, leading to horizontal widening. This lateral expansion exhibits cumulative effects in multi-layer stacking: as the number of printed layers increases, the Δ*H*, Δ*h*, and Δ*W* values generated in the lower layers propagate upward and amplify. Excessive merging leads to irregular structural deformation, resulting in a “dumbbell-shaped” morphology. The factors such as printing duration, process parameters, and three-dimensional architecture collectively contribute to Δ*h* values significantly exceeding the theoretical deformation Δ*H* in practical printing.

To further elucidate the dimensional evolution of printed gels, He et al. [74] conducted a systematic analysis of printed corners, mesh structures, and stacked layers. They introduced the horizontal spreading ratio (*φ*) to quantitatively assess the morphological evolution of mesh geometry and stacked structures, defined as:

(4) $$\varphi =\frac{A_{Rt}-A_{Re}}{A_{Rt}}$$

where *A_t_* represents the theoretical mesh area and *A_e_* denotes the experimentally measured area. Their results indicated that when printed meshes underwent irregular deformation and hydrogel at overlapping regions spread under gravity, *A_e_* become significantly smaller than *A_t_*, resulting in an increased φ. By enlarging the printing standoff distance (*D_L_*) to widen the gaps between dumbbell-shaped corners, the overlapping area can be effectively reduced, lowering φ and bringing the printed mesh closer to the intended geometric configuration (Figure 2f) [74]. Notably, the diffusion and coalescence of hydrogel not only decrease the printed layer height but also increase the line width, making the variations in height and width during structural evolution critical control parameters for maintaining dimensional fidelity in both horizontal and vertical directions. Individual deformation parameters (e.g., ΔH, ΔW) only capture dimensional changes in specific directions, whereas comprehensive geometric metrics (e.g., φ) can characterize the overall structural fidelity within a two-dimensional plane. Even so, these indicators remain primarily at the morphological level and fail to directly reflect the mechanical integrity of the structure. Therefore, a correlative analysis integrating these geometric metrics with rheological parameters (e.g., yield stress, creep recovery rate) and mechanical performance tests (e.g., compressive modulus) is essential to establish a comprehensive evaluation framework that bridges morphology and mechanical performance.

Building on the above mechanisms, we consolidate five key assessment dimensions: filament regularity, horizontal spreading, vertical merging, geometric fidelity of the mesh, and cumulative spreading effects across layers. As summarized in Table 2, systematic evaluation of the multi-layer stacking stability of different hydrogel systems can be achieved by quantifying deviations of parameters such as Δ*H*, Δ*h*, and Δ*W* from their theoretical values. Closer alignment with high-fidelity theoretical benchmarks indicates better performance in complex 3D fabrication. These analyzes provide important guidance for the development of hydrogel bioinks: on one hand, intrinsic stability can be improved through material modifications such as introducing dynamic crosslinks or optimizing rheological properties; on the other hand, process optimizations including print temperature control, spacing design, and crosslinking strategies can mitigate structural evolution during stacking. Such a material-process co-optimization approach establishes a methodological foundation for achieving high-precision and high-stability three-dimensional hydrogel constructs.

To systematically evaluate the printability and multi-layer stacking stability of hydrogel inks, the table presents quantitative characterization from five key dimensions: Line regularity is assessed by the ratio of theoretical to measured filament length; values closer to 1 indicate higher shape fidelity. Horizontal line spreading is quantified by the spreading ratio of measured filament width relative to the theoretical width; lower D values indicate better shape fidelity. Vertical line merging is evaluated using the layer height compression ratio, reflecting interlayer interface retention; lower F values represent higher shape fidelity. Mesh regularity is characterized by the printability index, which reflects pore geometric fidelity—P = 1 denotes an ideal square pore, while deviations indicate under- or over-gelation. Mesh line spreading is quantitatively described by the pore area loss rate, which measures material accumulation at intersection nodes; lower φ values indicate higher pore integrity.

## 4. Modeling and Prediction of Rheological Parameters and Printing Processes

The intricate interplay between rheological properties and printing outcomes necessitates the development of predictive models capable of accurately describing and optimizing the bioprinting process. Establishing quantitative relationships among material parameters, process variables, and printing fidelity provides a rational basis for bioink design and precise control of printing parameters [79,80]. This section examined both empirical and theoretical approaches for modeling extrusion behavior, with particular emphasis on the role of shear-induced effects in maintaining structural integrity and modulating cellular responses.

### 4.1. Empirical Models for Pressure-Modulus Relationships in Extrusion

The printing process of hydrogel inks encompasses three primary stages: extrusion, filament formation, and layer-by-layer stacking. Among these, extrusion pressure is a critical process parameter that governs both ink extrudability and the morphological fidelity of the printed structure [37,72,81]. To quantify the relationship between extrusion pressure and material rheology, Lee et al. [82] proposed a first-order iterative model that predicts the extrusion pressure (*Pr*) using the storage modulus (G′) and loss modulus (G″) as independent variables:

(5) $$P_{r}=\beta_{0}+\left(\beta_{1}\times G^{\prime }\right)+\left(\beta_{2}\times G^{\prime \prime }\right)+\left(\beta_{1}\times G^{\prime }\times G^{\prime \prime }\right)$$

where $\beta_{0}$, $\beta_{1}$, $\beta_{3}$, and $\beta_{3}$ are fitting constants.

Model analysis indicates that an increase in G″ leads to a rapid rise in extrusion pressure *Pr*. This is attributed to the simultaneous occurrence of high-frequency and low-frequency deformations during extrusion: in the high-frequency regime, the viscous response of the hydrogel exhibits solid-like behavior, and frequent deformation dominated by G″ significantly elevates extrusion resistance. Furthermore, the morphology of extruded filaments is closely related to the loss tangent (tanδ = G″/G′). A lower tanδ generally corresponds to irregular and rough filament contours, whereas a higher tanδ promotes the formation of smoother and more uniform extruded lines, thereby enhancing the geometric fidelity of the printed structure.

### 4.2. Theoretical Analysis of Shear Stress and Shear Rate Distribution Within Nozzles

**Figure 3:**
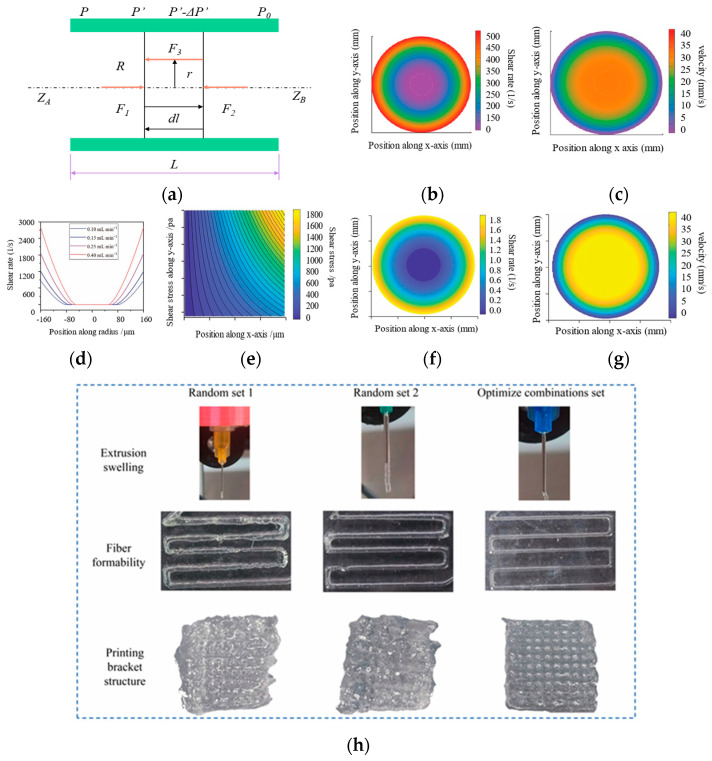
Rheological modeling and flow simulation of bioinks in a cylindrical nozzle. (**a**) Force-balance analysis of fluid flow in a circular tube. (**b**,**c**) Simulated shear-rate profile (**b**) and velocity profile (**c**) of the nanocomposite hydrogel bioink within the nozzle based on the Herschel-Bulkley fluid model [83]. (**d**) Dependence of wall shear rate and plug-flow diameter of the hydrogel microparticle bioink on extrusion rate [22]. (**e**) Shear stress profiles of the hydrogel microparticle bioinks within extruder tip [22]. (**f**,**g**) Predicted shear-rate profile (**f**) and velocity profile (**g**) of the hydrogel microparticle bioink under typical experimental printing conditions using the Herschel-Bulkley model [22]. (**h**) Scaffolds fabricated with different parameter combinations exhibited varying levels of structural integrity and distinct pore architectures [72]. (**b**,**c**) reprinted with permission from [83] Copyright© 2018 American Chemical Society. (**d**–**g**) reprinted with permission from [22] Copyright© 2020 Wiley Publishers. (**h**) reprinted from [72], under the CC BY 4.0 license.

During bioprinting, the distribution of shear stress and shear rate within the nozzle significantly influences the flow behavior of hydrogel inks, extrusion stability, and the viability of encapsulated cells. Given the challenges associated with direct measurement of the internal flow field, theoretical modeling based on fluid dynamics principles has become essential for analyzing stress distributions, optimizing printing parameters, and predicting cellular mechanical responses. A typical bioprinting nozzle can be simplified as a cylindrical pipe with a constant cross-section (Figure 3a). Under the assumption of steady laminar flow, fluid moves axially (along the z-direction) driven by a pressure gradient, exhibiting a parabolic velocity profile in the radial direction. Force balance analysis is performed on a fluid cylindrical element of radius (*r*) and length (*d_L_*). The forces acting on this element include the driving force due to the pressure difference across its ends, viscous resistance, and shear stress on its lateral surface. According to the force-balance equation:

(6) $$\sum F=F_{1}+F_{2}+F_{3}=0$$

which simplifies to:

(7) $$\pi r^{2}P^{\prime }-\pi r^{2}\left(P^{\prime }-{\Delta P}^{\prime }\right)-2\pi \tau_{r}dl=0$$

Rearranging yields the radial distribution of shear stress within the fluid:

(8) $$\tau_{r}=\frac{r}{2}\left(\frac{{\Delta P}^{\prime }}{dL}\right)$$

where Δ*P* is the pressure drop over the entire nozzle length *L*, and R is the nozzle radius. This expression indicates that shear stress varies linearly with radial position, reaching zero at the center (*r* = 0) and the maximum shear stress occurs at the nozzle wall (*r = R*):

(9) $$\tau_{wall}={∆P}^{\prime }\times \frac{R}{2L}$$

### 4.3. Rheological Models for Extrusion Behavior Analysis

Hydrogel bioinks typically exhibit shear-thinning behavior, which can be effectively described using either the power-law model or the Herschel-Bulkley model. The Herschel-Bulkley equation is expressed as:

(10) $$\tau =\tau_{0}+m\gamma^{n}$$

where τ represents the shear stress, $\tau_{0}$ is the yield stress (Pa), *m* denotes the consistency coefficient (·${Pa\cdot s}^{-1}$), γ is the shear rate ($s^{-1}$), and n is the flow behavior index. The shear stress at any radial position r can be expressed as:

(11) $$\tau =\tau_{0}+m\gamma^{n}=\frac{r}{R}\tau_{wall}=\frac{∆P^{\prime }}{2L}r$$

When the material exhibits no yield stress ($\tau_{0}=0$), the power-law model becomes a special case of the Herschel-Bulkley model. Fitting rheological data yields the parameters *m* and *n*, which allow calculation of the shear-rate distribution during ink extrusion through the nozzle. The shear-rate profile is given by:

(12) $$\gamma =\left\{\begin{array}{c} \;\;\;\;\;0\;\;\;\;\;\;\;\;\;\;\;\;\;\;\;\;\;\;\;\;\;\;\;\;\;\;\;\;\;\;\;\;\;\;\;\;\;\;\;\;\;\;\;\;\;\;\;\;0\leq r\leq R_{0}\;\\ {\left(\frac{\tau_{wall}}{m}\right)}^{\frac{1}{n}}{\left(\frac{r}{R}-\frac{\tau_{0}}{\tau_{wall}}\right)}^{\frac{1}{n}}\;{\;\;\;\;\;\;\;\;\;\;\;\;\;R}_{0}\leq r\leq R \end{array}\right.$$

Integration of the shear rate with respect to radius yields the velocity distribution (υ) across the nozzle:

(13) $$\upsilon =\left\{\begin{array}{c} R(\frac{n}{n+1}){\left(\frac{\tau_{wall}}{m}\right)}^{\frac{1}{n}}{\left(1-\frac{\tau_{0}}{\tau_{wall}}\right)}^{\frac{n+1}{n}}\;\;\;\;\;\;\;\;\;\;\;\;\;\;\;\;\;\;\;\;\;\;\;\;\;\;\;\;\;\;\;\;\;\;\;\;\;\;\;\;\;\;\;\;\;\;\;\;0\leq r\leq R_{0}\\ R\left(\frac{n}{n+1}\right){\left(\frac{\tau_{wall}}{m}\right)}^{\frac{1}{n}}\left({\left(1-\frac{\tau_{0}}{\tau_{wall}}\right)}^{\frac{n+1}{n}}-{\left(\frac{r}{R}-\frac{\tau_{0}}{\tau_{wall}}\right)}^{\frac{n}{n+1}}\right){\;\;\;\;\;\;\;\;\;\;\;\;\;R}_{0}\leq r\leq R \end{array}\right.$$

Finally, integration of the velocity profile over the nozzle cross-section gives the volumetric flow rate:

(14) $$Q=\pi R^{3}\left(\frac{n}{n+1}\right){\left(\frac{\tau_{wall}}{m}\right)}^{\frac{1}{n}}({\left(1-\frac{\tau_{0}}{\tau_{wall}}\right)}^{\frac{n+1}{n}}\left(1-\frac{2n}{\left(2n+1\right)\left(3n+1\right)}\left(1-\frac{\tau_{0}}{\tau_{wall}}\right)\left(\frac{\tau_{0}}{\tau_{wall}}n+2n+1\right)\right)$$

In the absence of yield stress ($\tau_{0}=0$), these expressions simplify to:

(15) $$\gamma ={\left(\frac{\tau_{wall}}{m}\right)}^{\frac{1}{n}}{\left(\frac{r}{R}\right)}^{\frac{1}{n}}$$

(16) $$\nu =R\left(\frac{n}{n+1}\right){\left(\frac{\tau_{wall}}{m}\right)}^{\frac{1}{n}}$$

(17) $$Q=\pi R^{3}\left(\frac{n}{3n+1}\right){\left(\frac{\tau_{wall}}{m}\right)}^{\frac{z}{n}}$$

These models provide a powerful tool for analyzing the extrusion process of hydrogel inks. Gaharwar et al. [83] developed a nanocomposite hydrogel system with measurable yield stress by integrating κ-carrageenan with methacrylated gelatin and nanoclay. They employed the Herschel-Bulkley model to examine the shear rate and velocity distributions during extrusion. When the yield stress of the hydrogel exceeds the applied shear stress, a solid-like plug flow region forms in the central part of the nozzle, where the velocity is uniform and no velocity gradient exists (Figure 3b,c) [83]. In our previous work, we developed a self-healing bioink based on pre-cross-linked chitosan methacrylate and polyvinyl alcohol hybrid hydrogel microparticles. The ink exhibits reversible gel-sol transition under shear, pronounced shear-thinning behavior, a yield stress of approximately 540 Pa, and rapid self-healing capability, making it suitable for extrusion-based 3D bioprinting. To gain deeper insight into its flow behavior during printing and the resulting influence on print quality, we performed numerical simulations using the Herschel-Bulkley model to analyze the shear-rate distribution and stress state of the microparticle system within the nozzle. Simulation results revealed a characteristic plug-flow profile inside the nozzle: the shear rate reached its maximum near the wall (up to 1800 s^−1^), while it approached zero in the central region, forming a nearly shear-free plug-flow zone (Figure 3d–g) [22]. This plug-flow region effectively shields the majority of the material from shear-induced damage, thereby enabling the direct printing of high-aspect-ratio and high-precision complex structures. Chai et al. [72] optimized the printing parameters based on mechanical properties to achieve consistent extrusion of hydrogel filaments without deposition delays. Random parameter combinations resulted in significantly compromised structural integrity due to poor filament formability, presenting a stark contrast to the superior performance of the optimized group. Scaffolds fabricated using random parameter combinations suffered from severe structural deformation caused by filament merging and collapse, failing to maintain three-dimensional integrity, with pore architecture becoming nearly indiscernible. In contrast, scaffolds produced with the optimized parameter combination exhibited excellent structural integrity and well-defined pore architecture. These results conclusively demonstrate the superior printing performance achieved with the optimized parameters, validating the scientific rigor and effectiveness of the parameter optimization methodology. Overall, by integrating rheological modeling with printing-process simulation, we established a theoretical framework for the rational design of bioinks and the systematic optimization of printing parameters. This approach advances 3D bioprinting from empirical trial-and-error toward a predictable and controllable methodology.

## 5. Comprehensive Evaluation Framework and Future Perspectives

The successful implementation of extrusion-based bioprinting relies on the synergistic optimization of multiple parameters, necessitating the establishment of a comprehensive evaluation framework that integrates rheological properties, structural fidelity, and biological performance [79,84,85]. This framework should incorporate quantitative metrics spanning from molecular-scale interactions to macroscopic structural integrity, enabling systematic correlation between material properties, processing parameters, and printing outcomes. Specifically, the evaluation system must encompass both static rheological parameters (e.g., zero-shear viscosity, equilibrium modulus) and dynamic response characteristics (e.g., shear-thinning exponent, recovery rate, gelation kinetics), coupled with quantitative analysis of printed structures through parameters such as line uniformity, grid regularity, and layer fusion quality [86,87,88]. The integration of these multi-scale indicators provides a robust foundation for rational bioink design and process optimization.

A persistent challenge in extrusion-based bioprinting lies in the inherent trade-off between optimal printability and favorable biological outcomes—two objectives often governed by conflicting material requirements. For instance, increasing polymer concentration or crosslinking density enhances shear-thinning behavior and shape fidelity but simultaneously reduces pore size and increases matrix stiffness, thereby restricting cell proliferation, migration, and matrix remodeling. Similarly, while a high yield stress improves structural self-support and filament fidelity, it inevitably elevates extrusion pressure and shear stress at the nozzle wall, leading to compromised cell viability and membrane integrity. Fast-gelling systems, although advantageous for preserving deposited geometry, may result in heterogeneous crosslinking or limited cellular reorganization.

It is worth emphasizing that quantitative rheological and structural metrics serve as a critical bridge connecting immediate post-printing cell survival with long-term tissue maturation. During the initial printing phase, parameters such as the shear-thinning index, yield stress, and shear recovery kinetics directly regulate the mechanical stress experienced by cells during extrusion and the subsequent structural integrity of the printed construct. Optimized shear-thinning behavior substantially mitigates cell membrane damage at the nozzle, while rapid shear recovery combined with an adequate storage modulus (G’) helps prevent structural collapse and maintain controlled inter-filament porosity. These factors collectively support immediate post-printing cell viability and facilitate the establishment of initial nutrient diffusion pathways. As culture progresses into the long-term phase, the biological relevance of these rheological and structural parameters becomes increasingly evident. Quantitative mechanical attributes—such as the equilibrium modulus after crosslinking and the stress relaxation rate—can be tailored to mimic the microenvironmental stiffness of specific native tissues, thereby guiding stem cell lineage specification (e.g., higher modulus favoring osteogenesis, faster stress relaxation supporting neuronal or adipogenic differentiation) [89]. Structural features—including microporosity interconnectivity, fiber alignment, and degradation kinetics, which are co-determined by yield stress and crosslinking behavior—provide a spatiotemporal framework for extracellular matrix deposition, cell migration, and tissue fusion [84]. These biomimetic characteristics ultimately govern key functional endpoints of tissue maturation, such as vascularization, specific protein expression, and synchronization of electromechanical activities (e.g., enhanced myocardial contractility, glycosaminoglycan accumulation in cartilage, or albumin secretion by hepatocytes). Thus, rheological and structural metrics not only define the processing window for predicting printability, but also delineate the potential trajectory from a three-dimensional biomimetic construct toward a fully functional tissue [90,91].

It should also be noted that while the Herschel-Bulkley model combined with laminar pipe flow provides a useful engineering approximation for predicting extrusion behavior, this theoretical framework inherently simplifies the complex flow characteristics of real bioinks. In practice, bioinks often exhibit non-ideal phenomena such as wall slip, time-dependent thixotropy, particle migration, and non-uniform crosslinking, factors which are not captured by idealized models. In this study, we critically examined these assumptions by drawing on the existing literature on the Herschel-Bulkley model and its application to complex fluids [92,93]. Regarding wall slip, the use of a specific nozzle geometry, coupled with experimental verification that slip velocity remained negligible within the investigated shear rate range, supports the validity of the no-slip assumption. This assumption yields conservative pressure drop estimates and is widely adopted in hydrogel extrusion modeling. Thixotropy measurements confirmed that the ink’s structural recovery rate substantially exceeded the printing timescale, thereby minimizing deviations from the steady-state flow behavior assumed by the Herschel-Bulkley model. With respect to particle or cell migration, the extremely short residence time within the nozzle during extrusion-based printing—combined with post-printing imaging—confirmed that cell distribution remained homogeneous, suggesting that sedimentation or shear-induced migration was not a significant concern in this system. Furthermore, it should be emphasized that the present model describes only the flow behavior of the pre-crosslinked ink prior to extrusion. Crosslinking occurs post-printing and therefore does not influence the fluid dynamics during the extrusion process itself, a distinction that is critical for accurately interpreting the rheological predictions in the context of biofabrication [92,94].

Recognizing that these trade-offs cannot be fully eliminated, we advocate for a paradigm shift from qualitative compromise toward quantitative trade-off management. Multi-objective optimization frameworks enable the identification of formulation and processing conditions that achieve an optimal balance between printability metrics (e.g., filament fusion ratio, shape fidelity index) and biological performance indicators (e.g., cell viability, proliferation, extracellular matrix deposition) [95,96]. Recent advances in dynamic covalent chemistry (e.g., Schiff base, Diels-Alder reactions) and supramolecular interactions (e.g., host-guest complexes, hydrogen bonding) offer promising pathways to decouple these traditionally conflicting attributes [86,97]. Furthermore, machine learning approaches are emerging as powerful tools for navigating the complex parameter space, enabling predictive optimization of bioink formulations and printing conditions based on historical data and theoretical models [98,99].

Given the current lack of unified data standards in the field of extrusion-based bioprinting, the deep integration of machine learning with experimental datasets does not necessarily depend on the a priori standardization of data. To address the modeling complexities inherent in non-structured data environments, a progressive integration strategy can be implemented through the following complementary pathways [100]. First, direct modeling based on locally consistent datasets has proven to be practically feasible. Even moderately sized rheological datasets (e.g., 100–200 samples) derived from a single experimental system, when combined with algorithms such as random forests or decision trees, can achieve high-precision predictions of key printability metrics (e.g., viscosity, filament width). This demonstrates that, even in the absence of cross-laboratory standardization, internally self-consistent datasets can still provide tangible support for the rational formulation optimization of bioinks [101]. Second, transfer learning offers a theoretically viable route to bridge heterogeneous data sources. By pre-training on large-scale material databases or rheological data from analogous polymer systems, followed by fine-tuning with limited experimental data from target bioink formulations, this approach can effectively mitigate the challenges of model generalization in small-sample regimes. Third, embedding domain knowledge as physical constraints into model architectures has emerged as a novel strategy for handling non-standardized data [80]. For instance, constructing composite loss functions based on thermodynamic consistency or classical Herschel-Bulkley constitutive models not only reduces reliance on massive training datasets but also enhances model interpretability and extrapolative capacity under diverse experimental conditions. Finally, the coupling of active learning with closed-loop bioprinting experimentation systems holds the potential to fundamentally transform the traditional “data first, modeling later” research paradigm. By iteratively selecting the most informative experimental conditions in real time, such systems can generate structured, high-value datasets as an integral part of the printing process, thereby enabling the co-evolution of machine learning modeling and process optimization. In summary, data standardization and the application of machine learning are not sequential stages in a linear progression, but rather a mutually reinforcing, dual-track process of co-evolution. The former lays the foundation for long-term model generalizability and the robust accumulation of data, while the latter already provides practically applicable predictive tools for rational formulation design in the present [79,102].

A hierarchical evaluation system should be established, incorporating both material-centric and process-centric parameters. At the material level, key indicators include: (1) shear-thinning index (n) reflecting extrusion ease, (2) yield stress (τ_0_) determining structural self-supporting capability, (3) recovery time (t_re_c) characterizing self-healing efficiency, and (4) gelation time (t_gel_) governing solidification kinetics [32,33]. At the structural level, quantitative metrics such as printing accuracy ratio, line diffusion rate, and layer fusion index provide objective assessment of printing fidelity. The integration of these indicators into a standardized scoring system would significantly facilitate comparative analysis between different bioink systems and accelerate material development cycles [80,103,104].

Future bioink development should focus on multi-functional systems that simultaneously address printability, biocompatibility, and bioactivity. This includes: (1) incorporating enzyme-responsive or pH-sensitive moieties for targeted drug release in pathological microenvironments [105,106,107], (2) designing interpenetrating networks combining rapid physical gelation with covalent crosslinking for enhanced mechanical stability [108], and (3) integrating bioactive peptides to promote specific cellular responses [109]. Particularly, stress-yielding hydrogels that exhibit solid-like behavior under low stress yet flow under high stress represent an ideal platform for protecting cells during extrusion while maintaining structural integrity post-printing. Additionally, the development of in situ monitoring systems coupled with closed-loop feedback control could dynamically adjust printing parameters based on real-time assessment of filament formation and cell viability [3,110,111,112].

## 6. In Summary

The advancement of three-dimensional bioprinting reflects a paradigm shift from empirical optimization to quantitatively driven and standardized methodologies. This review systematically synthesizes an integrated evaluation framework for hydrogel printability, founded on three interconnected pillars: rheological characterization, microstructural analysis, and theoretical modeling. The dynamic rheological properties of hydrogels, including shear-thinning behavior, yield stress, self-healing capability, and creep resistance, collectively establish the physical foundation for extrusion feasibility, structural retention, and long-term stability. Quantitative assessment of printed structures using metrics such as the printability factor and line diffusion rate provides reproducible and objective standards for comparative evaluation of bioinks and printing processes. Furthermore, theoretical models such as the Herschel-Bulkley and extrusion pressure models enable the correlation of material parameters with shear fields and flow profiles during printing, thereby facilitating the prediction and control of critical process variables such as wall shear stress that directly influence structural fidelity and cellular integrity.

Future advancements in bioprinting will hinge on multidisciplinary and multi-scale innovation. A central priority is the development of intelligent bioinks that integrate dynamic covalent bonds and supramolecular chemistries to exhibit stimuli-responsive, self-healing, and stress-yielding characteristics. Such functionalities synergistically enhance printability, cytocompatibility, and biological performance. Equally critical are standardization initiatives and data-driven methodologies, including unified rheological testing protocols, benchmarking of printing accuracy, and open-access bioink databases coupled with machine learning, which collectively accelerate the optimization of bioink formulations and printing parameters. The implementation of closed-loop biomanufacturing systems incorporating in situ monitoring and real-time feedback control will further ensure dimensional precision and cell viability across scales, from individual filaments to complex volumetric tissues. By establishing a rigorous quantitative foundation and fostering cross-disciplinary integration, bioprinting is poised to overcome existing limitations in structural complexity, functional maturity, and manufacturing reproducibility. These advances will expedite the clinical translation of bioprinted constructs into tangible applications in regenerative medicine, disease modeling, and drug screening.

## Figures and Tables

**Table 1:** Rheological properties of hydrogel bioinks.

Materials	Viscosity	Yield Stain/Stress	Self-Healing	Creep	Ref
Pre-crosslinked hydrogel microparticle inks: chitosan methacrylate/polyvinyl alcohol	778–23,522 Pa·s	5%	Recovery from 10 Pa to 250 Pa	0.35% deformation	[22]
Monounsaturated glucolipid G-C18:1	10–10^5^ Pa·s	5.6–210 Pa	Recovery from 10 Pa to 2000 Pa	N/A	[23]
hyaluronic acid, calcium hydroxyapatite	50 Pa·s	15.85%	100%	0%	[24]
Microfibrillated cellulose, gelatin methacryloyl, sodium alginate	7.7–7967 Pa·s	491 ± 9 Pa	92%	N/A	[25]
Gelatin Glycidyl Methacrylate, Sodium alginate, cellulose	1000–10,000 Pa·s	1%	Recovery from 10 Pa to 250 Pa	Structure stable at temperatures < 26 °C	[26]
Methacrylated collagen peptide, xanthan gum	1000–10,000 Pa·s	111%	Rapid, reversible self-healing ability	N/A	[27]
Nanofibrillated cellulose, Fucose-rich polysaccharide	1491.7 ± 41.5 Pa·s	102.9 ± 5.9 Pa	99.8 ± 3.0%	N/A	[6]
Gelatin Methacrylate, Alginate, carboxymethyl cellulose	4.6 Pa·s	132 ± 25 Pa	100%	N/A	[28]
Polyacrylamide, N,N’-Methylenebisacrylamide, konjac glucomannan	Exhibits shear-thinning behavior	0.285 MPa	Recovery from 1800 Pa to 18,000 Pa	N/A	[29]
GelMA, HA-NB, Elastin	47,770.1 mPa·s	19.85 kPa	N/A	GHE hydrogel exhibits complete shape recovery	[30]
Alginate, nanocellulose	10,000–20,000 Pa·s	20–50 Pa	80%	N/A	[31]
Yeast protein, alginate, xanthan gum	1,481,684 ± 8091 mPa·s	15.4 ± 0.5%	85 ± 6%	N/A	[32]
Gelatin, Sodium alginate, carbohydrazide	12–1000 Pa·s	242.1 kPa	60.8%	N/A	[33]
Fmoc-FFY, MCC, alginate	600 Pa·s	45%	100%	N/A	[34]
Fmoc-FF, Sodium Alginate	80–80,000 Pa·s	39.173 ± 13.637 Pa	Excellent self-healing ability	N/A.	[35]
PEG, GelMA, Fibrinogen, Nanosilicates	0.1–500 Pa·s	112 Pa	80%.	N/A	[36]
Sodium Alginate, Gelatin	485–2460 Pa·s	4688.56 Pa	N/A	N/A	[37]
κ-carrageenan	5–10,000 Pa·s	20%	Excellent self-healing ability	N/A	[38]
PVA, FBA	275–510 Pa·s	N/A	93.9%	N/A	[39]
CS, DFPEG, SA,	Exhibits shear-thinning behavior	5%	100%	N/A	[40]

**Table 2:** Quantitative evaluation of ink printability based on the structure morphology.

Assessment Parameters	Quantitative Analysis	Quantitative Range
Line regularity	$R=\frac{L_{t}}{L_{e}}$	0 < R ≤ 0.1
		0.1 < R ≤ 0.5
		0.5 < R ≤ 0.9
		0.9 < R ≤ 1.0
Horizontal line spreading	$D=\frac{w_{e}-w_{t}}{w_{t}}=\frac{∆w}{w_{t}}$	D ≥ 0.5
		0.3 < D ≤ 0.5
		0.1 < D ≤ 0.3
		0 ≤ D ≤ 0.1
Vertical line merging	$F=\frac{h_{e}-h_{t}}{h_{t}}=\frac{∆h}{h_{t}}$	0.3 < F ≤ 0.5
		0.1 < F ≤ 0.3
		0 ≤ F ≤ 0.1
Mesh regularity	$P=\frac{\pi }{4}\cdot \frac{1}{C}=\frac{L^{2}}{16A_{e}}$	P < 0.9
		P > 1.1
		0.9 ≤ P ≤ 1.1
Mesh line spreading	$\varphi =\frac{A_{t}-A_{e}}{A_{t}}\times 100\%$	φ ≥ 0.5
		0.3 < φ ≤ 0.5
		0.1 < φ ≤ 0.3
		0 ≤ φ ≤ 0.1

## Data Availability

Figure 2a reprinted from [72], under the CC BY 4.0 license. Figure 2b reprinted from [73], under the CC BY 4.0 license. Figure 2d–f reprinted from [74], under the CC BY 4.0 license. Figure 3b,c reprinted with permission from [83] Copyright© 2018 American Chemical Society. Figure 3d–g reprinted with permission from [22] Copyright© 2020 Wiley Publishers. Figure 3h reprinted from [72], under the CC BY 4.0 license.
